# Unveiling the diversity, composition, and dynamics of phyllosphere microbial communities in *Alhagi sparsifolia* across desert basins and seasons in Xinjiang, China

**DOI:** 10.3389/fmicb.2024.1361756

**Published:** 2024-03-25

**Authors:** Yulin Zhang, Yi Du, Zhihao Zhang, Waqar Islam, Fanjiang Zeng

**Affiliations:** ^1^College of Ecology and Environmental, Xinjiang University, Urumqi, China; ^2^State Key Laboratory of Desert and Oasis Ecology, Key Laboratory of Ecological Safety and Sustainable Development in Arid Lands, Xinjiang Institute of Ecology and Geography, Chinese Academy of Sciences, Urumqi, China; ^3^Xinjiang Key Laboratory of Desert Plant Roots Ecology and Vegetation Restoration, Xinjiang Institute of Ecology and Geography, Chinese Academy of Sciences, Urumqi, China; ^4^Cele National Station of Observation and Research for Desert-Grassland Ecosystems, Cele, China; ^5^University of Chinese Academy of Sciences, Beijing, China

**Keywords:** seasonal dynamics, phyllosphere microbiome, climate effects, co-occurrence patterns, desert ecosystems

## Abstract

Phyllosphere microbes residing on plant leaf surfaces for maintaining plant health have gained increasing recognition. However, in desert ecosystems, knowledge about the variety, composition, and coexistence patterns of microbial communities in the phyllosphere remains limited. This study, conducted across three basins (Turpan-TLF, Tarim-CL, and Dzungaria-MSW) and three seasons (spring, summer, and autumn) in Xinjiang, China, aimed to explore the diversity and composition of microbial communities in the phyllosphere, encompassing both bacteria and fungi in *Alhagi sparsifolia*. We also investigated the co-occurrence patterns, influencing factors, and underlying mechanisms driving these dynamics. Results indicate that phyllosphere bacteria exhibited lower diversity indices (ACE, Shannon, Simpson, Fisher phylogenetic diversity, and Richness) in spring compared to summer and autumn, while the Goods Coverage Index (GCI) was higher in spring. Conversely, diversity indices and GCI of phyllosphere fungi showed an opposite trend. Interestingly, the lowest level of multi-functionality and niche width in phyllosphere bacteria occurred in spring, while the highest level was observed in phyllosphere fungi. Furthermore, the study revealed that no significant differences in multi-functionality were found among the regions (CL, MSW, and TLF). Network analysis highlighted that during spring, phyllosphere bacteria exhibited the lowest number of nodes, edges, and average degree, while phyllosphere fungi had the highest. Surprisingly, the multi-functionality of both phyllosphere bacteria and fungi showed no significant correlation with climatic and environmental factors but displayed a significant association with the morphological characteristics and physicochemical properties of leaves. Structural Equation Model indicated that the morphological characteristics of leaves significantly influenced the multi-functionality of phyllosphere bacteria and fungi. However, the indirect and total effects of climate on multi-functionality were greater than the effects of physicochemical properties and morphological characteristics of leaves. These findings offer new insights into leaf phyllosphere microbial community structure, laying a theoretical foundation for vegetation restoration and rational plant resource utilization in desert ecosystems.

## 1 Introduction

Plant leaves serve as a diverse and expansive habitat for microbial colonization, hosting a myriad of species across the globe’s approximately 300,000 plant varieties, each characterized by unique physical structures, leaf chemistries, and ecological distributions (Mora et al., 2011; Finkel et al., 2016). The phyllosphere, encompassing both epiphytic and endophytic microorganisms, presents a distinct environment from the rhizosphere, with larger apoplasts in leaves facilitating gas exchange for photosynthesis and offering an air-filled habitat for microbial colonization (Lindow and Brandl, 2003; Chen et al., 2020). Microorganisms in the phyllosphere play an important role in maintaining plant health and even ecosystem functions. Desert ecosystem is a common ecosystem type in arid and semi-arid areas, which is limited by extreme environmental conditions such as extreme lack of water, high temperature, and poor nutrients (Ibáñez et al., 2007; Zhang et al., 2020; Tariq et al., 2022). Desert plants adapt to these extreme conditions through special adaptation mechanisms (Gao et al., 2022; Lin et al., 2023). *Alhagi sparsifolia* is a typical desert phreatophyte with a deep root system, widely distributed in arid and salinized areas of central Asia, where it plays a vital role in maintaining the structure and function of the ecosystems and promoting regional animal husbandry development (Zhang et al., 2020, 2022; Tariq et al., 2022). The in-depth study of the phyllosphere microbial community characteristics of *A. sparsifolia* is of great practical significance for a comprehensive understanding of the overall ecological adaptation mechanism, to effectively protect and rationally utilize plant resources.

Recent research focuses on the connection between microbial biodiversity and ecosystem multi-functionality (Hector and Bagchi, 2007; Gamfeldt and Roger, 2017). However, the impact of leaf morphology, functional traits, and climatic factors on this relationship remains understudied in desert ecosystems. This study addresses these gaps by employing CoNet (Co-occurrence network), diversity, multi-functionality, and niche width inference to elucidate the diversity, composition, and co-occurrence networks of microbial communities in the phyllosphere of *A. sparsifolia* across three distinct basins (Turpan Basin-TLF, Tarim Basin-CL, and Dzungaria Basin-MSW) and three seasons (spring, summer, and autumn) in Xinjiang, China. The research aims to discern variations in microbial communities within the phyllosphere of *A. sparsifolia* across different basins, identify ecological factors influencing diversity, composition, and network features, and assess potential variations among habitats, regions, and microbial phyla (bacteria and fungi). We aimed to address the following questions: (i) Are the diversity, composition, and co-occurrence patterns of microbial communities of leaf phyllosphere of *A. sparsifolia* different in the three basins? (ii) What ecological factors drive the diversity, composition, and network topological features, and do these ecological drivers and their contribution differ between habitats (leaf phyllosphere), regions (Turpan Basin, Tarim Basin, and Dzungaria Basin), and phylum (bacteria and fungi)? We hypothesized that seasonal and regional changes will affect the diversity, multi-functionality, niche width, and network complexity of leaf phyllosphere microorganisms of three basins. Moreover, we wanted to know, whether the phyllosphere microorganisms (bacteria and fungi) show complementary effects in seasonality. The research will provide novel insights into the structure of leaf phyllosphere microbial communities, establishing a theoretical foundation for vegetation restoration and the rational utilization of plant resources in desert ecosystems.

## 2 Materials and methods

### 2.1 Experimental site

**Figure 1:**
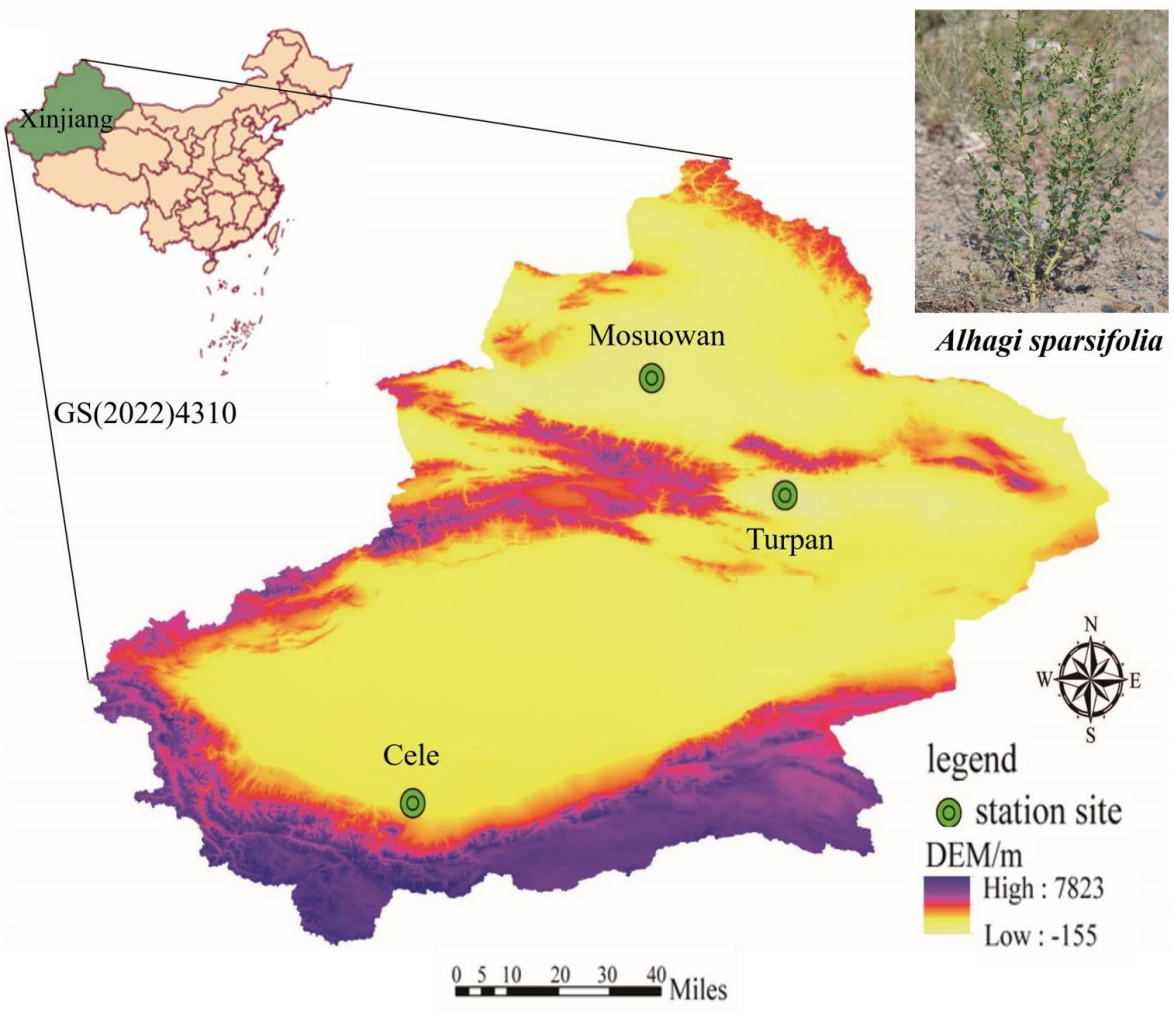
The three sampling sites at Cele, Turpan, and Mosuowan are located in Tarim Basin, Turpan Basin, and Junggar Basin, respectively.

**Figure 2:**
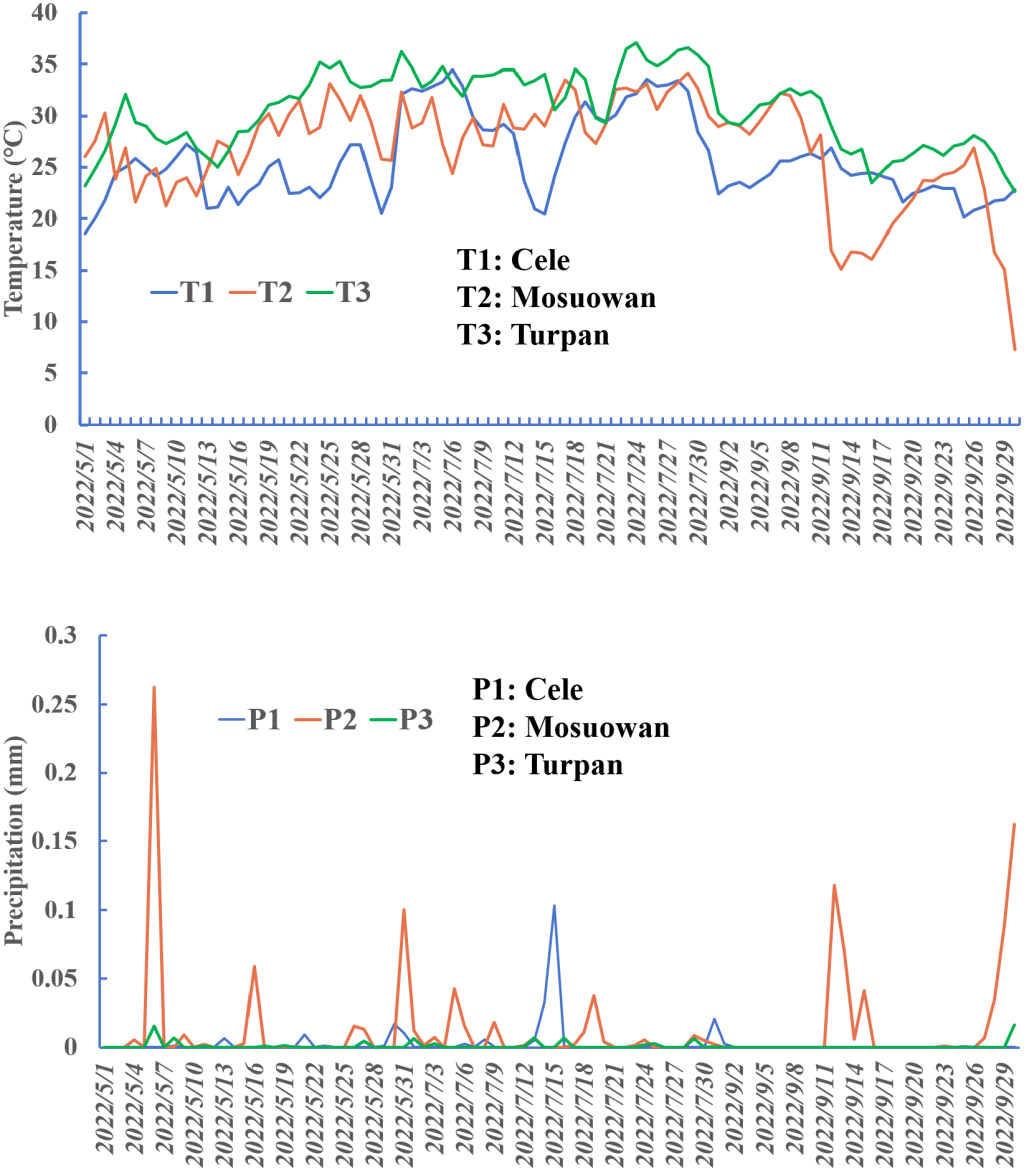
Precipitation and temperature plots at sampling points.

The experiment was conducted at three desert stations, namely: (a) Cele Desert Research Station (CL): Located on the southern border of the Tarim Basin at coordinates 37°00′57″N, 80°43′45″E, with an altitude of 1,318 m. The average yearly temperature is 11.9°C, annual precipitation averages 35.1 mm, and potential evaporation amounts to 2595.3 mm. There are approximately 20 days per year when dust storms occur, and dust is present for a total of 240 days. (b) Turpan Desert Botanical Garden (TLF): Situated in the arid expanse of Central Asia, within the Eurasian hinterland, at coordinates 42°51′N, 89°11′E. This botanical garden is unique as it is located at an elevation ranging from −105 m to −76 m, making it the world’s lowest. The region receives an average annual rainfall of 16.4 mm, while the annual evaporation rate is 3,000 mm. The mean yearly temperature is recorded as 13.9°C, and the frost-free period spans 265.6 days. (c) Mosuowan Desert Research Station (MSW): Positioned at 45°01′N, 89°11′E, this station is characterized by a continental arid climate. It sits at an altitude of 346 m, with an average yearly temperature of 6.6°C. Precipitation averages around 117 mm annually, and evaporation measures approximately 1979.5 mm. A frost-free period extends from 135 to 205 days (Figures 1, 2).

### 2.2 Sampling design

For each of the three study fields, encompassing CL, TLF, and MSW, a total of four homogeneous quadrats were chosen. Each quadrat, measuring approximately 30 m × 30 m, featured thriving natural populations of the desert plant *A. sparsifolia*, serving as representative samples. In total, 12 research blocks were selected, distributed across the three study fields. Throughout the spring, summer, and autumn of 2022, comprehensive sampling was undertaken from the phylloplane and leaf endosphere at these well-established, long-term, *in-situ* observation stations (No samples were collected in winter because the leaves of the plants were all dead). Stringent hygiene practices were adopted during the sampling process, with researchers wearing masks and sterile gloves. Disease-free leaves were chosen from all cardinal directions (east, west, north, and south) of *A. sparsifolia* within each quadrat, with a total of 60 leaves. Sterile swabs were used to clean the leaves, and subsequently, the cleaned leaves were carefully placed into sterile centrifuge tubes. Extra leaves from each cardinal direction were stored separately to maintain sample integrity. To prevent potential cross-contamination, gloves were changed, and hands were sanitized using alcohol-soaked cotton balls between each sampling instance. Upon arrival at the laboratory, the swabs and leaves within the centrifuge tubes were temporarily stored in an icebox maintained at a temperature of 4°C. During this period, they underwent pre-treatment for a duration of 2 to 5 h. This pre-treatment involved immersing the leaves in 95% alcohol, oscillating them for three cycles of 15 s each. Subsequently, the samples underwent three washes with sterile water and were placed in sterile centrifuge tubes at −80°C for the extraction of leaf endosphere bacteria.

Each set of samples comprised four biological replicates, resulting in a total of 72 samples collected throughout 2022, accounting for two compartments (phylloplane and leaf endosphere), three basins (CL, TLF, and MSW), three seasons, and four replicates. Genomic DNA extraction procedures were performed, and the resultant samples were stored at −80°C for subsequent analysis.

### 2.3 DNA extraction, PCR, Illumina sequencing, and bioinformatics analysis

To extract complete genomic DNA from 500 mg of phylloplane and leaf endosphere, the DNeasy Power Soil DNA Isolation Kit (Qiagen, Inc., Netherlands) was employed, adhering to the guidelines provided by the manufacturer. PCR (Polymerase Chain Reaction) amplification of bacterial 16S rRNA genes V3-V4 region and fungal ITS 1–5 (Internal Transcribed Spacer) region was performed using universal primers 341 (5′-CCTAYGGGRBGCASCAG-3′) and 806 R (5′-GGACTACNNGGGTATCT AAT-3′); ITS 5-1737F (5′-GGAAGTAAAAGTCGTAACAAGG-3′) and ITS 2-2043R (5′-GCTGC GTTCTTCATCGATGC-3′), respectively (Nossa et al., 2010). Amplicon quality was determined with gel electrophoresis, purified with Agencourt AM-Pure XP beads, and amplified PCR (only one round). Qubit dsDNA assay kit (Life Technologies, United States) was used to quantify the final amplicon. Equal amounts of purified amplicon were pooled and sequenced using the Illumina HiSeq X Ten, PE150 platform.

The Uparse software (v7.0.1001) was used to generate operational taxonomic units (OTUs) with a 97% similarity threshold (Edgar, 2013). The QIIME (v1.91) was used to select the representative read for each out (Caporaso et al., 2010). Bacterial OTUs were classified using the RDP classifier (with a confidence threshold of 70%) according to either the Silva database (version 132) or Greengenes (Wang et al., 2007). To ensure bacterial and fungal (leaf phyllosphere) sequence uniformity among all samples, the minimum number (36,551 and 38,560) of sequences was used as the depth to filter other samples to generate in a filtered ASV table {This result removed plant sequences (“mitochondria “, “chloroplasts”) in fungal results, however, bacteria do not remove plant sequences}. The raw sequencing data are available at the National Center for Biotechnology Information (NCBI) Short Read Archive, Bio Project ID PRJNA1024038.

### 2.4 Measurement of leaf physicochemical properties and morphological characteristics

The leaf organic carbon (LOC) was determined using the K_2_Cr_2_O_7_-H_2_SO_4_ oxidation method, and total nitrogen (TN) concentration was measured using the Kjeldahl Nitrogen Analyzer (K1160, Jinan Hanon Instruments Co. Ltd., China) (Bao, 2000). After digestion in concentrated HNO_3_, the alkali hydrolyzable method was used to measure the available nitrogen (AN), while the Inductively Coupled Plasma-Optical Emission Spectrometer (iCAP 6,300, Thermo Elemental, United States) was employed to determine the total phosphorus (TP) and total potassium (TK) (Hooper and Vitousek, 1998). Available phosphorus (AP) was extracted using HCl/NH_4_F through colorimetric analysis on a continuous-flow autoanalyzer (Autoanalyzer 3, Bran and Luebbe, Germany) employing ascorbic acid molybdate (Lu, 1999). To determine the availability of potassium (AK), the NH_4_OAc extraction technique was utilized (Warra et al., 2015). Using a pH and EC meter (PHSJ-6 L and DDSJ-319 L, respectively) made by INESA Scientific Instrument Co. Ltd. in China, the acidity level and electrical conductivity of the leaf were determined. The pH was measured with a soil/water ratio of 1:2.5 (w/v), while the EC was measured with a soil/water ratio of 1:5 (w/v).

The leaf area was determined by employing the ImageJ program (Softonic International, Spain) following the established protocol. Afterward, the samples were placed in an oven at a temperature of 75°C for 24 h to determine their leaf dry weight (LDW). The specific leaf area (SLA) was calculated by dividing the leaf area by the LDW. However, the specific leaf weight (SLW) was calculated by dividing the LDW by the leaf area (Zhang et al., 2020). The physiochemical properties and morphological characteristics of leaves are shown in Supplementary Table S1.

### 2.5 Metrological/climate data

The experiment collected meteorological data including the temperature, air relative humidity, atmospheric pressure, precipitation, wind speed, wind direction, direct radiation, indirect radiation, and diffuse radiation from the National Meteorological Science Data Center (http://data.cma.cn/) at the CL, TLF, and MSW of the Chinese Academy of Sciences (Supplementary Table S2).

### 2.6 Assessing multi-functionality

The phylloplane and leaf endosphere were combined into phyllosphere microorganisms for analysis. To obtain a quantitative multi-functionality of the phyllosphere (bacteria and fungi) of *A. sparsifolia* from the different seasons (spring, summer, and autumn) and regions (CL, TLF, and MSW), two distinct and complementary multi-functionality approaches were used: (i) the average multi-functionality approach provides an easy-to-interpret and straight forward measure of the ability of an ecosystem to simultaneously sustain multiple functions, and this approach is widely used by current multi-functionality studies, (ii) the assessment of multi-dimensional functioning through the extraction of fundamental axes of ecosystem variation from principal coordinate analysis, serving as a representation of overall ecosystem functioning (Manning et al., 2018; Delgado-Baquerizo et al., 2020). The following calculation methods were used:

(i) Selected eight α-diversity indexes that were closely related to species diversity (Observed, Chao1, ACE, Shannon, Simpson, InvSimpson, Fisher phylogenetic diversity, and Goods coverage) to represent multi-functionality. First of all, these α-diversity indexes are normalized, and then their Z-scores are calculated (Maestre et al., 2012). The calculation formula is:


(1) $$Z=\left(x_{i}-\lambda_{i}\right)/\delta_{i}$$

Where *Z* is the Z-score of the α-diversity index, and *x_i_* is the value of the α-diversity index parameter; *λ_i_* is the average value of the α-diversity index. *δ_i_* is the standard deviation of the α-diversity index. Averaging the Z-score of α-diversity is called average multi-functionality (Maestre et al., 2012). The calculation formula is:


(2) EMF=∑i8xi8

(ii) The α-diversity indices (Observed, Chao1, ACE, Shannon, Simpson, InvSimpson, Fisher phylogenetic diversity, and Goods coverage) were normalized and principal component analysis was performed. The interpretation rate of the first two axes is greater than 90%. Therefore, the first two axes are selected to evaluate the multi-functionality.

### 2.7 Statistical analyses

The statistical analysis was performed using R4.1.0 (R Core Team, 2021). The phylloplane and leaf endosphere were combined into phyllosphere microorganisms for analysis. To evaluate the influence of season and sampling location on α-diversity measures (Richness, ACE, Shannon, Simpson, Fisher phylogenetic diversity, and Goods coverage), a one-way analysis of variance (ANOVA) was utilized. The 1% level was used to determine statistical significance, and *p*-values were adjusted by applying the false discovery rate (FDR) (Benjamini et al., 2006). To identify the sources of variation, the agricolae package’s LSD test function was utilized (Mendiburu, 2020). The niche width of bacteria and fungi in the phyllosphere was calculated using the Spaa package (method = Levins) (Jiao et al., 2020), and then visualized using the ggplot2 package (Ginestet, 2011). The Mantel test with 999 permutations was employed to assess Spearman’s correlations between the multi-functionality of bacteria and fungi and various factors, including α-diversity, leaf physicochemical properties, morphological characteristics, and climate. Additionally, using the vegan package (Oksanen et al., 2017), redundancy analysis (RDA) models were utilized to determine the significant influence of leaf physicochemical properties, morphological characteristics, and climate factors on different phyllosphere bacteria and fungi at the phyla-level communities. The structural equation model (SEM) implemented in Amos-24 software was utilized to investigate the causal relationships among climate factors, leaf physicochemical properties, morphological characteristics, bacteria and fungi richness, and phyllosphere bacterial and fungal multi-functionality. Additionally, the Co-occurrence networks were constructed in Gephi,^1^ using a Spearman’s correlation matrix with an absolute correlation coefficient (r > 0.7) and FDR-adjusted (*p* < 0.001).

## 3 Results

### 3.1 Variation characteristics of phyllosphere microbial diversity

**Figure 3:**
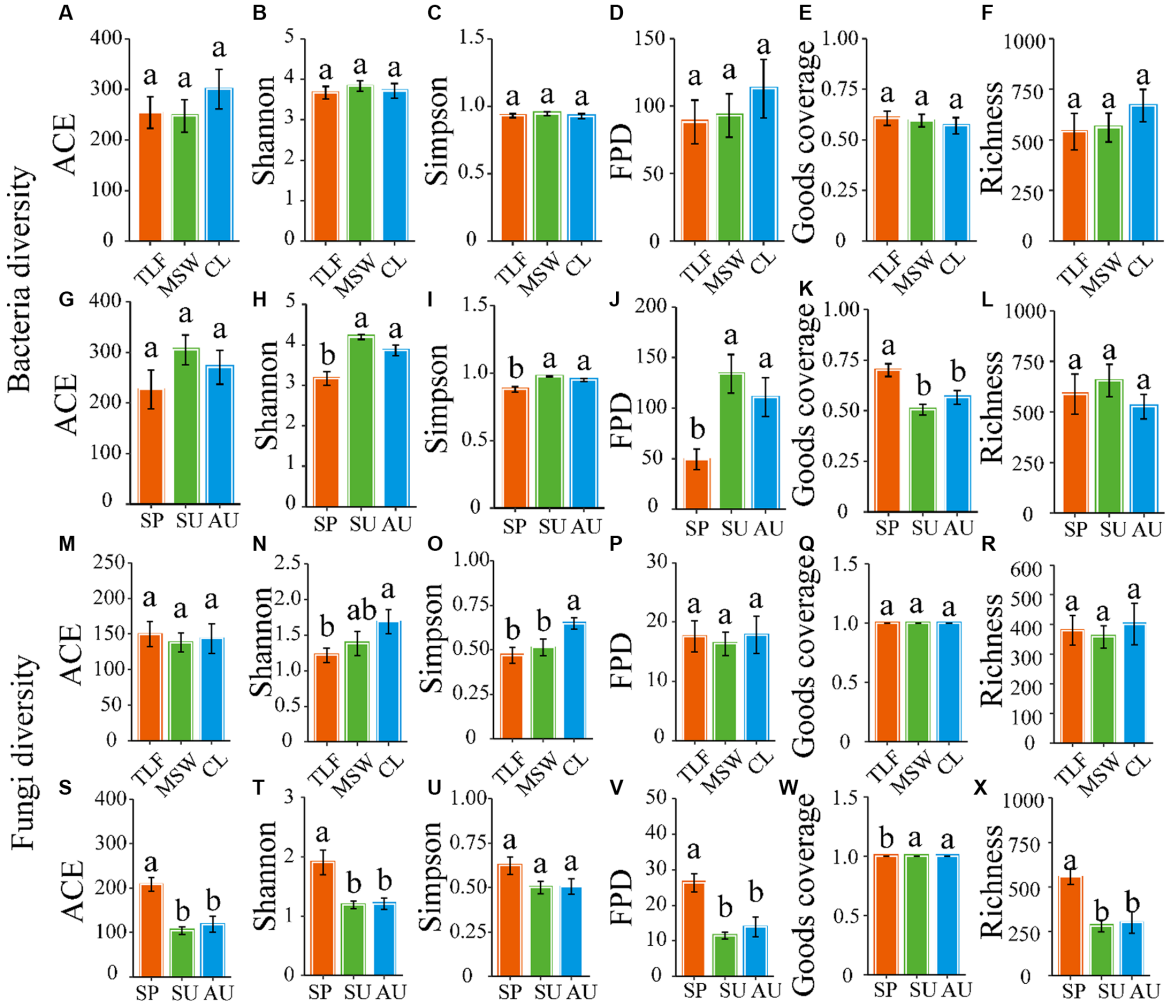
Seasonal changes of phyllosphere microbial community diversity. Seasonal changes of microbial community diversity [**A** and **G** (ACE), **B** and **H** (Shannon), **C** and **I** (Simpson), **D** and **J** (Fisher phylogenetic diversity), **E** and **K** (Goods coverage), **F** and **L** (Richness)] of the phyllosphere bacteria, and [**M** and **S** (ACE), **N** and **T** (Shannon), **O** and **U** (Simpson), **P** and **V** (Fisher phylogenetic diversity), **Q** and **W** (Goods coverage), **R** and **X** (Richness)] of the phyllosphere fungi. Different lowercase letters indicate significant differences among elevations at the *p* < 0.05 level (ANOVA and Duncan’s test). When comparing different seasons, samples from different locations were mixed into one group. When comparing different sites, samples from different seasons are taken as a group. CL, Cele; MSW, Mosuowan; TLF, Turpan; SP, spring; SU, summer; AU, autumn; FPD, Fisher phylogenetic diversity.

The α-diversity of phyllosphere bacteria, as measured by various indices including ACE, Shannon, Simpson, Fisher, Coverage, and Richness, did not exhibit significant differences among three distinct regions (CL, MSW, and TLF) (Figures 3A–F; *p* > 0.05). Moreover, the ACE and Richness indices showed no significant variation across spring, summer, and autumn (Figures 3G, L; *p* > 0.05). The phyllosphere bacteria exhibited notably decreased values for the Shannon, Simpson, and Fisher phylogenetic diversity during the spring season when compared to both summer and autumn (Figures 3H–J; *p* < 0.05). In spring, the Goods coverage index (GCI) of phyllosphere bacteria showed notably higher values compared to both summer and autumn (Figure 3K; *p* < 0.05).

The ACE, Fisher, Coverage, and Richness indexes of phyllosphere fungi exhibited no significant differences among the three typical regions (CL, MSW, and TLF) in terms of diversity (Figures 3M,P,Q,R; *p* > 0.05). However, the Shannon and Simpson indexes of phyllosphere fungi were significantly higher in CL compared to TLF (Figures 3N,O; *p* < 0.05). Additionally, the Simpson index of phyllosphere fungi did not show any significant variations across spring, summer, and autumn (Figure 3U; *p* > 0.05). In spring, the phyllosphere fungi exhibited significantly higher ACE, Shannon, Fisher phylogenetic diversity, and Richness indexes compared to summer and autumn (Figures 3S,T,V,X; *p* < 0.05). In contrast, the phyllosphere fungi exhibited a notably decreased GCI during the spring season in comparison to both summer and autumn (Figure 3W; *p* < 0.05).

### 3.2 Multi-functionality of phyllosphere microorganisms

**Figure 4:**
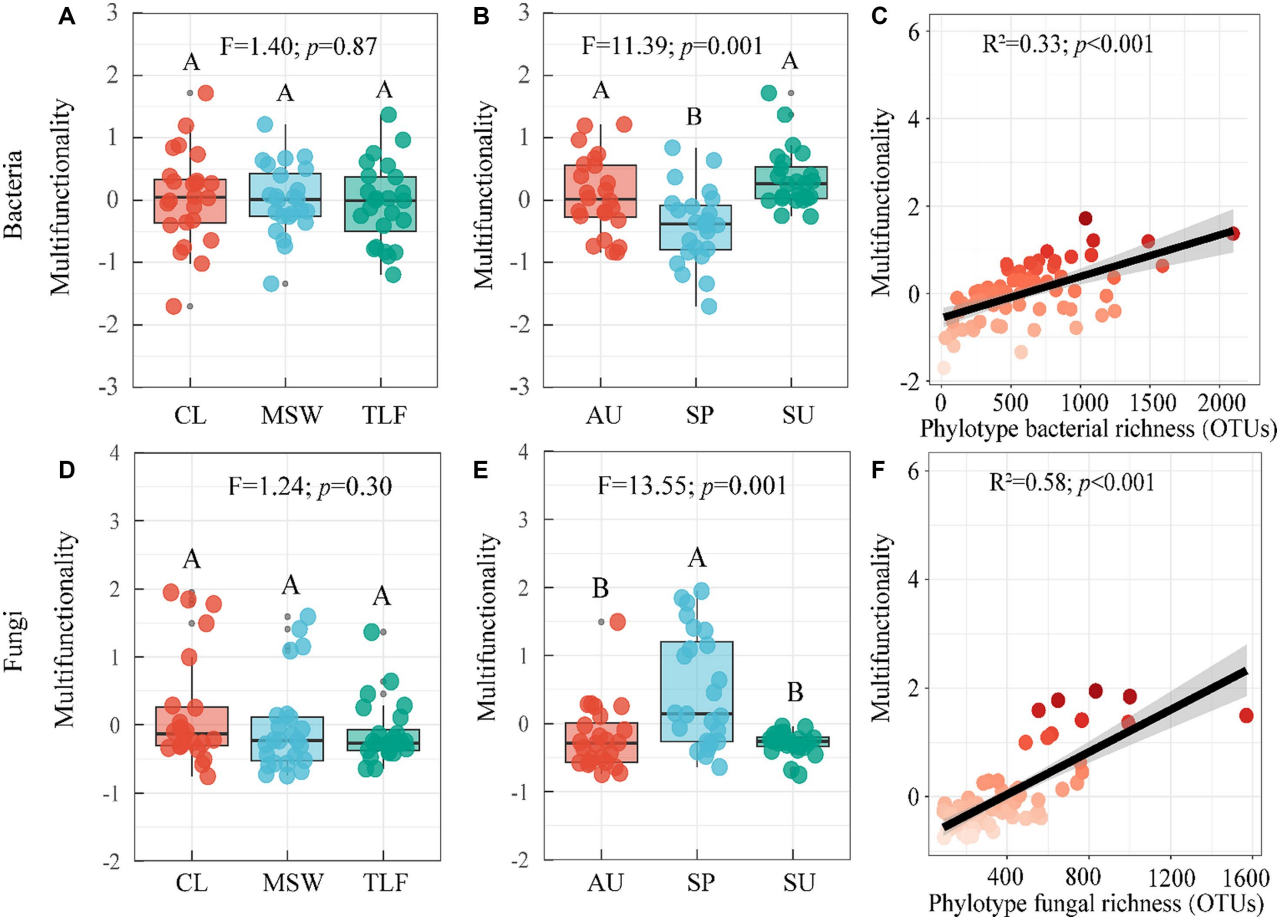
Seasonal changes of phyllosphere microbial community multi-functionality. [**A**, **B**, and **C** (phyllosphere bacterial multifunctionality)] and [**D**, **E**, and **F** (phyllosphere fungal Multifunctionality)]. Different lowercase letters indicate significant differences among elevations at the *p* < 0.05 level (ANOVA and Duncan’s test). The differences between groups between sites were also compared (*p* < 0.05). When comparing different seasons, samples from different locations were mixed into one group. When comparing different sites, samples from different seasons are taken as a group. CL, Cele; MSW, Mosuowan; TLF, Turpan; SP, spring; SU, summer; AU, autumn.

The multifunctionality of phyllosphere bacteria and fungi does not exhibit significant differences across the three typical regions (CL, MSW, and TLF) (Figures 4A,D; *p* > 0.05). During spring, the multi-functionality of phyllosphere bacteria was significantly lower compared to summer and autumn, while the multi-functionality of phyllosphere fungi displayed the opposite pattern (Figures 4B,E; *p* < 0.05). Furthermore, with the increase in richness, the multi-functionality of phyllosphere bacteria and fungi increased, indicating robust stability (Figures 4C,F; *p* < 0.001) (Equations 1, 2).

**Figure 5:**
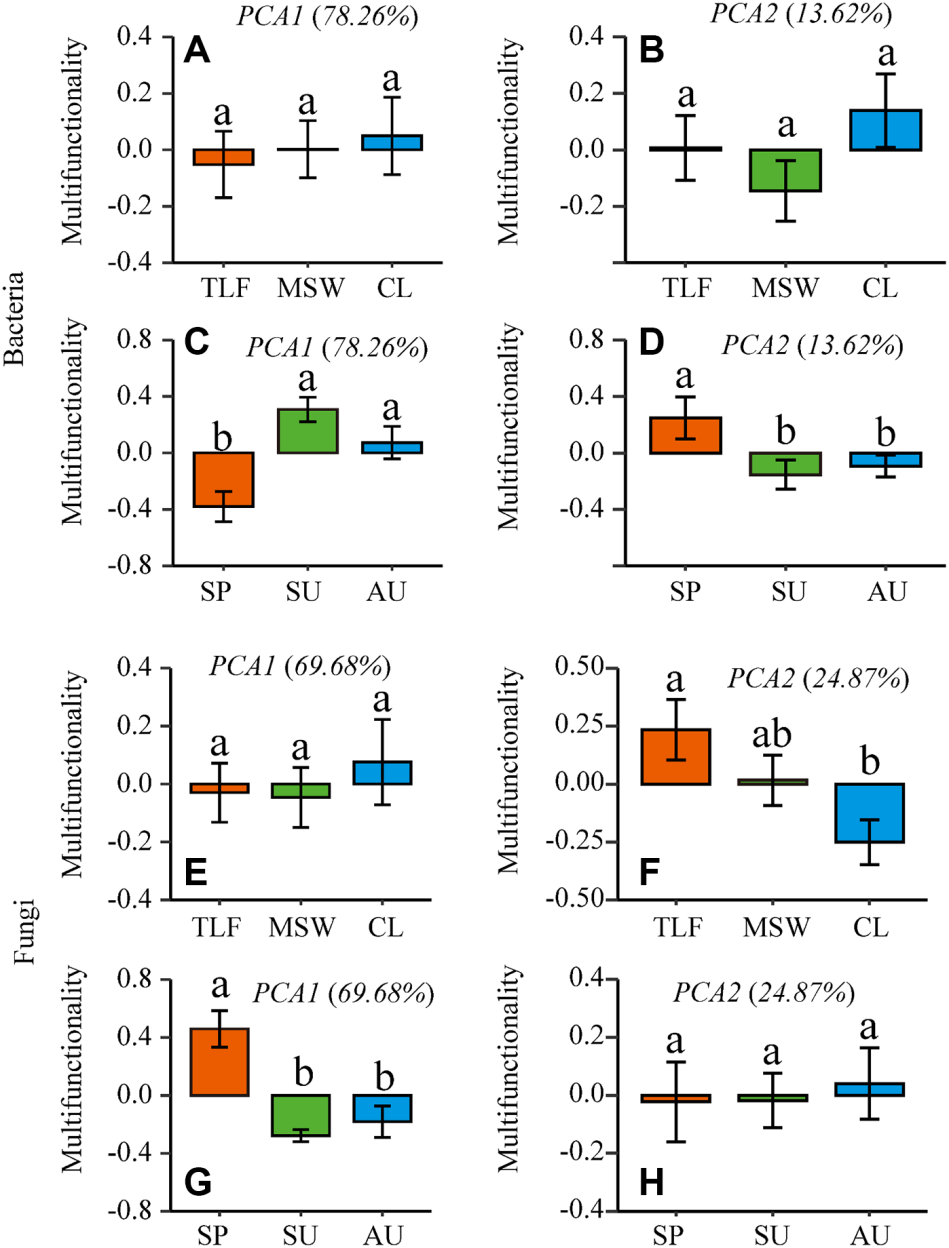
The first two dimensions (PCA1-2) of the multidimensional multi-functionality [**A**, **B**, **C**, and **D** (phyllosphere bacterial multifunctionality)] and [**E**, **F**, **G**, and **H** (phyllosphere fungal multifunctionality)] were selected by principal component analysis (PCA). When comparing different seasons, samples from different locations were mixed into one group. When comparing different sites, samples from different seasons are taken as a group. CL, Cele; MSW, Mosuowan; TLF, Turpan; SP, spring; SU, summer; AU, autumn.

The first two axes of multi-functionality were selected by principal component analysis (PCA). In terms of the multifunctionality exhibited by phyllosphere bacteria, no significant differences were observed across three representative regions (CL, MSW, and TLF) (Figures 5A,B; *p* > 0.05). It was discovered that the multi-functionality of phyllosphere microorganisms in the primary dimension was diminished in spring in contrast to summer and autumn, whereas the multi-functionality in the secondary dimension was elevated in spring as opposed to summer and autumn (Figures 5C,D; *p* < 0.05). No significant variation in the multifunctionality of phyllosphere fungi was detected across the three typical regions (CL, MSW, and TLF) on the first axis and different season (spring, summer, and autumn) on the second axis (Figures 5E,H; *p*  > 0.05). Moreover, the multi-functionality of phyllosphere fungi in the second axis was significantly lower in CL compared to TLF (Figure 5F; *p* < 0.05). Furthermore, the multi-functionality of phyllosphere fungi in the first axis exhibited higher values during spring as opposed to summer and autumn (Figure 5G; *p* < 0.05).

### 3.3 Co-occurrence patterns and niche width of phyllosphere microorganisms

**Figure 6:**
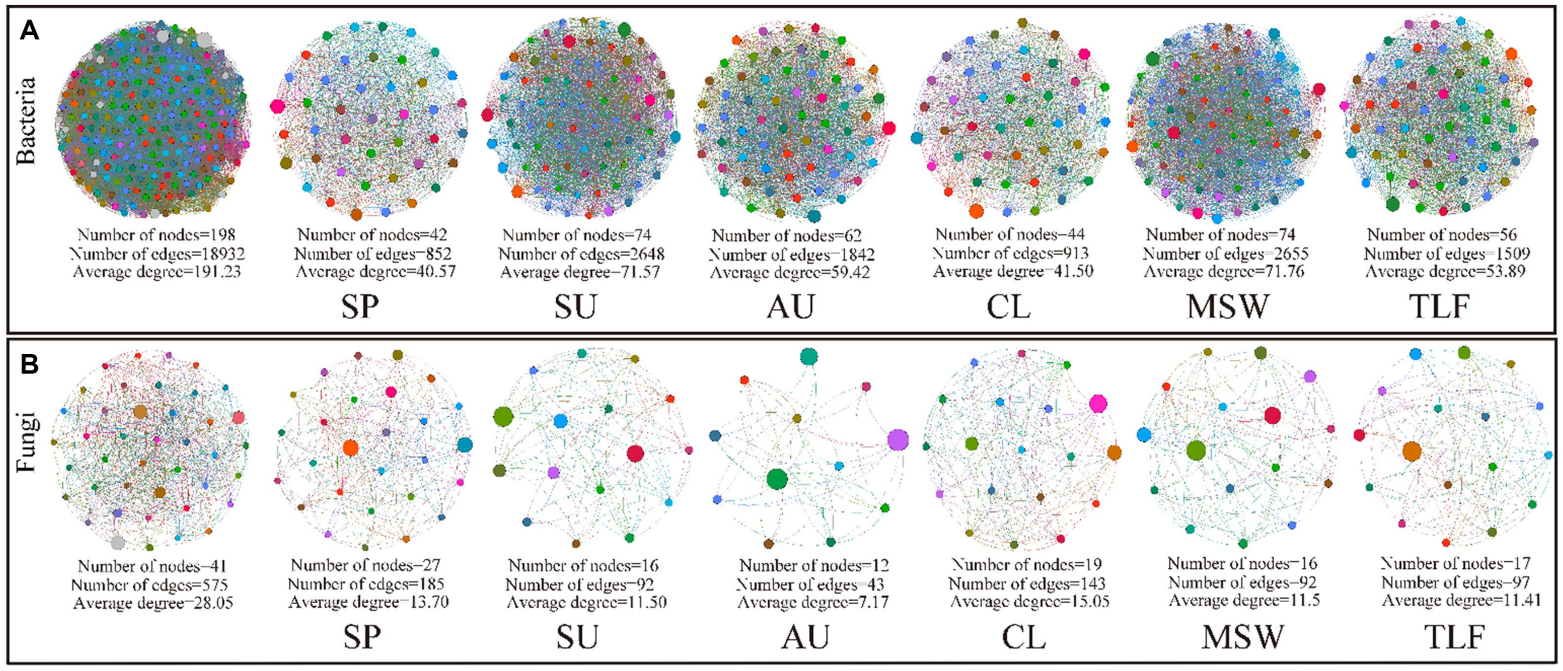
Network co-occurrence model of phyllosphere microbial community [**A** (phyllosphere bacterial network)] and [**B** (phyllosphere fungal network)]. We mixed the bacteria and fungi in the phylloplane and leaf endosphere, respectively. In different seasons and regions, a bacterial network was constructed using 8(4 leaf phylloplane+4 leaf endosphere) replicas, as was the fungal network. CL, Cele; MSW, Mosuowan; TLF, Turpan; SP, spring; SU, summer; AU, autumn.

The network analysis conducted on phyllosphere bacteria and fungi across various seasons and regions revealed that during spring, the nodes, edges, and average degree of phyllosphere bacteria were comparatively lower than those observed in summer and autumn, and that of phyllosphere fungi showed the opposite trend. Moreover, in the MSW region, the nodes, edges, and average degree of phyllosphere bacteria were higher than those observed in the CL and TLF regions, but phyllosphere fungi showed the opposite trend. The network structure of phyllosphere bacteria exhibits greater complexity in various seasons or regions compared to that of fungi. In particular, in the springtime, the lowest number of positive and negative connections between the borders of phyllosphere bacteria is noticed, while the highest number of positive and negative connections between the borders of phyllosphere fungi is observed (Figures 6A,B; Supplementary Table S3).

**Figure 7:**
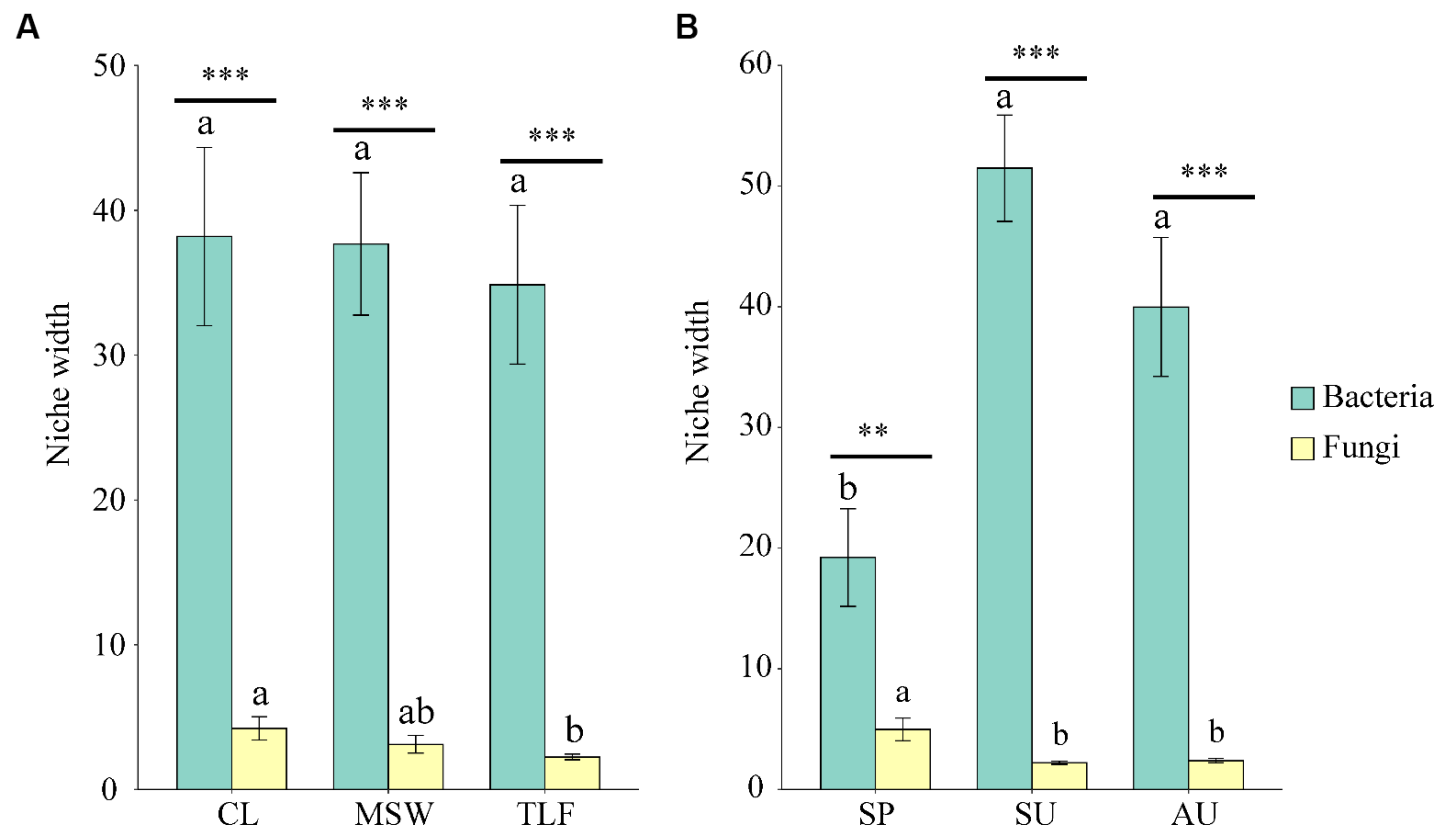
Seasonal and regional changes of phyllosphere microbial niche width [**A** and **B** (phyllosphere bacterial and fungal niche width)]. Different lowercase letters indicate significant differences among elevations at the *p* < 0.05 level (ANOVA and Duncan’s test). The bacteria and fungi were performed by t.test function in different seasons (SP, SU, and AU) and regions (CL, MSW, and TLF). When comparing different seasons, samples from different locations were mixed into one group. When comparing different sites, samples from different seasons are taken as a group. CL, Cele; MSW, Mosuowan; TLF, Turpan; SP, spring; SU, summer; AU, autumn. Significance codes, “**,” *p* < 0.01; “***,” *p* < 0.001.

The niche width of phyllosphere bacteria did not exhibit significant differences among three distinct regions (CL, MSW, and TLF) (Figure 7A; *p* > 0.05). In contrast, the niche width of phyllosphere fungi was significantly higher in CL compared to TLF (Figure 7A; *p* < 0.05). In spring, the niche width of phyllosphere bacteria notably lower values compared to both summer and autumn (Figure 7B; *p* < 0.05), however, fungi showed the opposite trend.

### 3.4 Mental test of multi-functionality of phyllosphere microorganisms and plant traits and environmental factors

**Figure 8:**
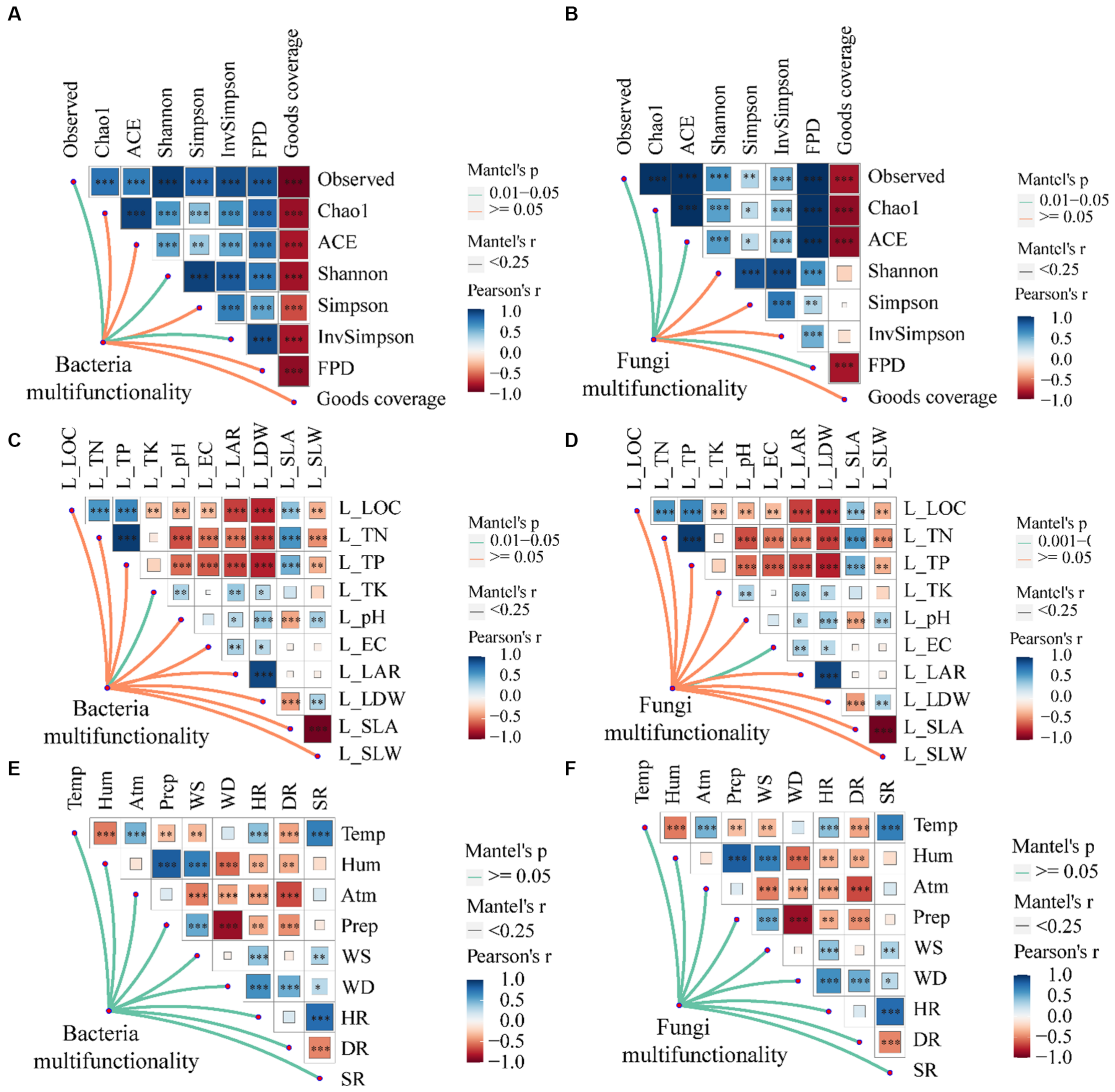
The relationship between leaf microbial community multi-functionality [**A**, **C**, and **E** (phyllosphere bacterial multifunctionality)] and [**B**, **D**, and **F** (phyllosphere fungal multifunctionality)] and leaf morphological characteristics, physical and chemical properties, climate, and environmental factors. LOC, leaf organic carbon (g⋅kg^−1^); TN, total nitrogen (g⋅kg^−1^); TP, total phosphorus (g⋅kg^−1^); TK, total potassium (g⋅kg^−1^); EC, electrical conductivity (mS⋅cm^−1^); LAR, leaf area (cm^2^); LDW, leaf dry weight (g); SLA, specific leaf area (cm^2^⋅g^−1^); SLW, specific leaf weight (g⋅cm^−2^). Temp, temperature (°C); Hum, humidity (%); Atm, atmospheric pressure (hPa); Prep, precipitation (mm); WS, wind speed (m⋅s^−1^); WD, wind direction (°); HR, horizontal radiation(w⋅m^−2^); DR, direct radiation(w⋅m^−2^); SR, scattered radiation (w⋅m^−2^); FPD, Fisher phylogenetic diversity. Significance codes, “*,” *p* < 0.05; “**,” *p* < 0.01; “***,” *p* < 0.001.

There exists a noteworthy association between the multi-functionality of phyllosphere bacteria and α-diversity indexes such as observed, Shannon, and Inv Simpson, as well as a significant correlation with the total potassium content of leaves (Figures 8A,C; *p* < 0.05). Similarly, the multi-functionality of phyllosphere fungi demonstrates a significant correlation with observed, Chao1, ACE, and Fisher phylogenetic diversity, along with a significant correlation with leaf electrical conductivity (Figures 8B,D; *p* < 0.05). However, the multi-functionality of both phyllosphere bacteria and fungi does not exhibit a significant correlation with climatic factors (Figures 8E,F; *p* > 0.05).

### 3.5 Redundancy analysis of the diversity of phyllosphere microorganisms and plant traits and environmental factors

**Figure 9:**
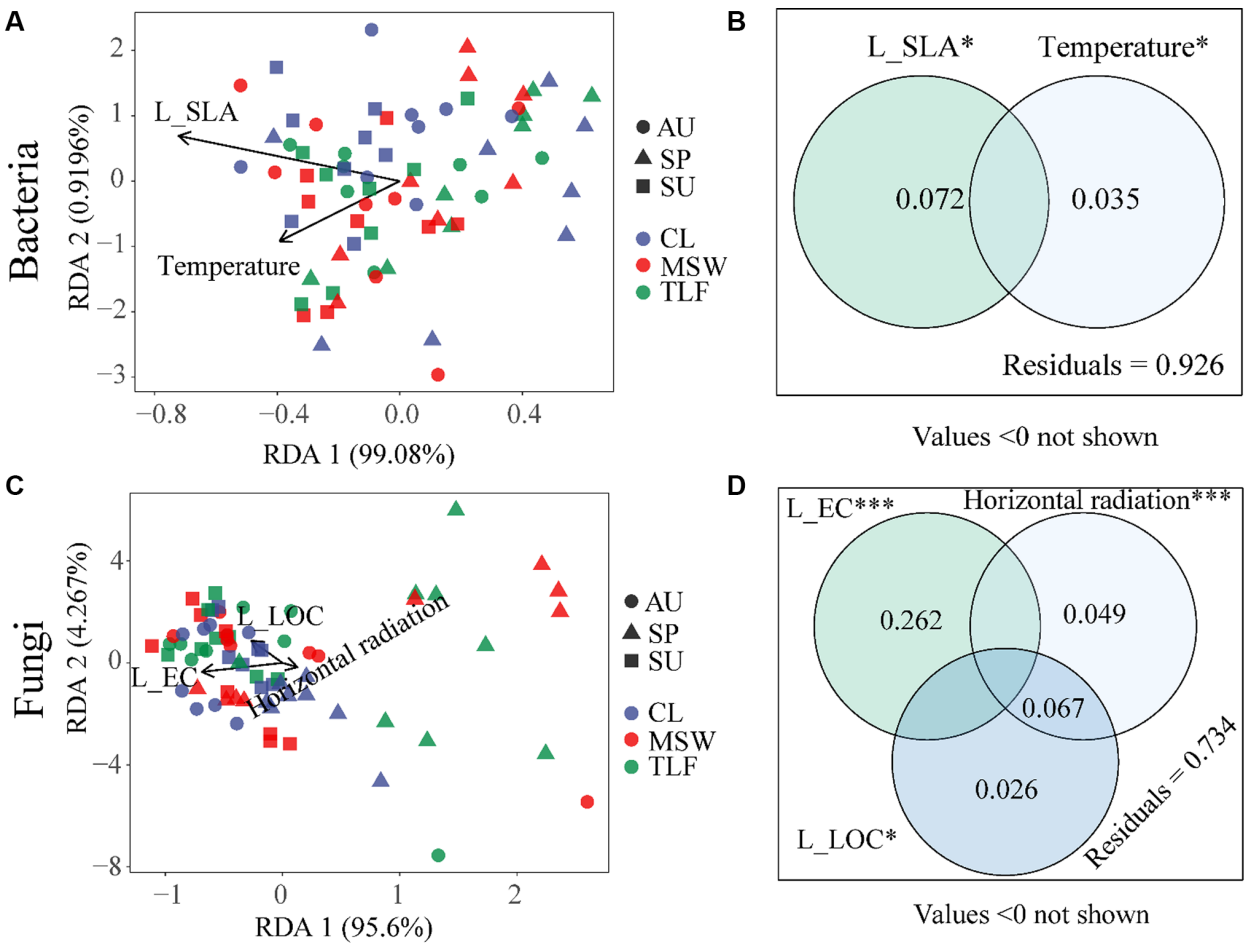
The relationship between leaf microbial diversity [**A** and **B** (influencing factors of phyllosphere bacterial diversity)] and [**C** and **D** (influencing factors of phyllosphere fungal diversity)] and leaf morphological characteristics, physicochemical properties, climate, and environmental factors. When comparing the factors influencing the diversity of bacteria and fungi separately, samples from different locations and seasons were mixed into one group. CL, Cele desert; MSW, Mosuowan desert; TLF, Turpan desert; SP, spring; SU, summer; AU, autumn. LOC, leaf organic carbon (g⋅kg^−1^); EC, electrical conductivity (mS⋅cm^−1^); SLA, specific leaf area (cm^2^⋅g^−1^). Significance codes, “*,” *p* < 0.05; “***,” *p* < 0.001.

Redundancy Analysis (RDA) revealed the influential factors on microbial communities extracted from a total of 10 leaves and 9 climate parameters. The analysis showed that the diversity of phyllosphere bacteria displayed clustering patterns during various seasons at the three sampling locations, suggesting a greater degree of multi-functionality (Figure 9A). In contrast, the diversity of phyllosphere fungi was more scattered, especially in the spring (Figure 9C). The diversity of phyllosphere bacteria was notably affected by L-SLA (*p* < 0.05, *R*^2^ = 0.072) and temperature (*p* < 0.05, *R*^2^ = 0.035; Figure 9B). The diversity of phyllosphere fungi was greatly affected by L-EC (*p* < 0.001, *R*^2^ = 0.262), L-LOC (*p* < 0.05, *R*^2^ = 0.093), and horizontal radiation (*p* < 0.001, *R*^2^ = 0.116; Figure 9D). Moreover, during the spring, a direct relationship was observed between horizontal radiation and the diversity of phyllosphere fungi (Figure 9C). The results indicate that the influence of leaf morphological characteristics and climatic conditions on the diversity of phyllosphere fungi is more significant compared to phyllosphere bacteria (Figures 9A–D).

### 3.6 Structural equation model analysis of the multi-functionality of phyllosphere microorganisms and plant traits and environmental factors

**Figure 10:**
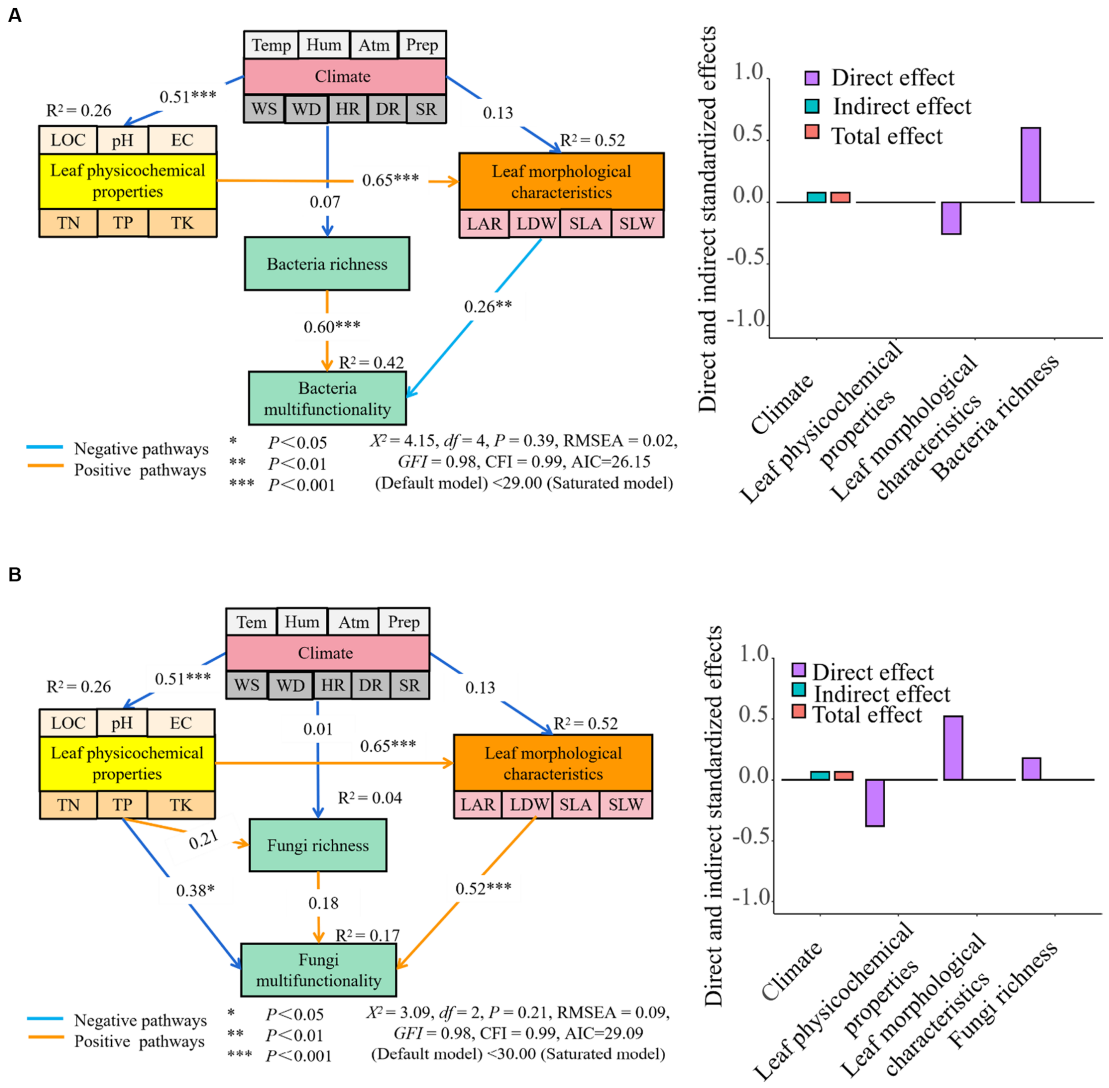
The path analysis between the multi-functionality of leaf microbial community [**A** (phyllosphere bacteria multifunctionality path analysis) and **B** (phyllosphere fungi multifunctionality path analysis) and leaf morphological characteristics, physicochemical properties, and climatic environmental factors. LOC, leaf organic carbon (g⋅kg^−1^); TN, total nitrogen (g⋅kg^−1^); TP, total phosphorus (g⋅kg^−1^); TK, total potassium (g⋅kg^−1^); EC, electrical conductivity (mS⋅cm^−1^); LAR, leaf area (cm^2^); LDW, leaf dry weight (g); SLA, specific leaf area (cm^2^⋅g^−1^); SLW, specific leaf weight (g⋅cm^−2^). Temp, temperature (°C); Hum, humidity (%); Atm, atmospheric pressure (hPa); Prep, precipitation (mm); WS, wind speed (m⋅s^−1^); WD, wind direction (°); HR, horizontal radiation (w⋅m^−2^); DR, direct radiation(w⋅m^−2^); SR, scattered radiation (w⋅m^−2^). Significance codes, “*,” *p* < 0.05; “**,” *p* < 0.01; “***,” *p* < 0.001.

The findings from the analysis of SEM demonstrated that a combination of climate, leaf physicochemical properties, leaf morphological characteristics, and bacterial richness together explained 42% of the variations in multi-functionality observed in phyllosphere bacteria. Leaf morphological characteristics and bacterial richness were found to have a direct impact on the multi-functionality of phyllosphere bacteria. Furthermore, the influence of climate on the multi-functionality of phyllosphere bacteria was found to be indirect, mediated through alterations in the physicochemical properties and morphological characteristics of the leaf. The impact of leaf morphological characteristics and bacterial richness on the multi-functionality of phyllosphere bacteria was greater than that of climate and leaf physicochemical properties. The indirect and total effects of climate on the multi-functionality of phyllosphere bacteria were observed to be greater than those of leaf physicochemical properties, leaf morphological characteristics, and bacterial richness (Figure 10A).

However, the climate, leaf physicochemical properties, leaf morphological characteristics, and fungal richness together explain 17% of the multi-functionality changes of phyllosphere fungi. Specifically, the physicochemical properties and morphological characteristics of leaves, as well as the richness of fungi, directly impact the multi-functionality of phyllosphere fungi. Additionally, climate indirectly affects the multi-functionality of phyllosphere fungi by altering the physicochemical properties and morphological characteristics of the leaf. The impact of leaf physicochemical properties, leaf morphological characteristics, and fungal richness on the multi-functionality of phyllosphere fungi was greater than that of climate. Conversely, the indirect and total effects of climate on the multi-functionality of phyllosphere fungi were greater than physicochemical properties, morphological characteristics, and fungal richness of the leaf (Figure 10B).

Finally, the potential influence of leaf physicochemical properties on the multi-functionality of phyllosphere bacteria may be ignored, and the negative direct effect on the multi-functionality of phyllosphere fungi is greater. Leaf morphological traits exhibited a positive direct impact on the multi-functionality of phyllosphere bacteria, albeit to a lesser extent than the positive direct impact on the multi-functionality of phyllosphere fungi (Figures 10A,B).

## 4 Discussion

The intricate interplay between the diversity and complexity of phyllosphere microorganisms is linked to their multifaceted roles. This relationship manifests in their capacity to enhance the absorption of plant nutrients, optimize the intricate functions of microbial flora, and improve the morphological characteristics of plants (Philippot et al., 2013; Vacher et al., 2016; Xiong et al., 2021a,b). The investigation reveals substantial disparities in the size and composition of phyllosphere microorganism populations, both spatially and temporally, and even within the same plant species across different locations (Jackson et al., 2006; Bonito et al., 2014; Bashir et al., 2022; Perreault and Laforest-Lapointe, 2022; Wei et al., 2022).

Notably, the diversity indices of phyllosphere bacteria in spring were lower compared to summer and autumn, with a higher GCI in spring. Compared to summer and autumn, in spring, it was found that the bacterial niche width was lowest, whereas the fungal niche width was highest. However, phyllosphere fungi exhibited higher diversity indices in spring and lower Goods coverage indices compared to summer and autumn. This intriguing observation underscores a pronounced complementarity between bacterial and fungal diversity in the phyllosphere of the desert deep-rooted plant (*A. sparsifolia*), suggesting a potential niche complementarity effect. This aligns with the “niche complementarity hypothesis,” emphasizing that diverse trait values enhance ecosystem functioning (Petchey and Gaston, 2006; Diaz et al., 2007).

Analyzing the diversity and multi-functionality of phyllosphere microorganisms (especially across seasons and regions) can potentially enhance resource availability, plant photosynthesis, nutrient synthesis, and nutrient utilization (Kembel et al., 2014; Purahong et al., 2016; Chen et al., 2020). However, the study uncovered no significant differences in the diversity of phyllosphere microorganisms among three representative regions (CL, MSW, and TLF), except for the notably higher diversity of phyllosphere fungi in CL. This emphasizes strong geographical influences on phyllosphere fungi, indicating a robust locality effect. Previous research has similarly highlighted greater geographic distinctiveness and dispersal limitations in phyllosphere fungi compared to bacteria (Peay et al., 2007; Vacher et al., 2016; Abdelfattah et al., 2021; Wei et al., 2022). Geographical factors, such as temperature and rainfall, in Turpan and Cele impact the composition of the phyllosphere microorganism community, showcasing the multi-functionality and variety of its microorganisms.

Bacteria and fungi engage in functional promotion, antagonism, and various chemical-mediated mechanisms during their interactions (Schmidt et al., 2019). The simultaneous consideration of bacterial and fungal communities enables a comprehensive evaluation of their complementary effects on ecosystem function (Soliveres et al., 2016). The study found the multi-functionality of phyllosphere bacteria to be lowest and that of fungi to be highest in spring. However, multi-functionality did not significantly differ among the three typical regions (CL, MSW, and TLF), indicating that seasonal changes have a more pronounced impact than geographical regions.

This investigation further supports the notion that phyllosphere microorganisms associated with the deep-rooted desert plant (*A. sparsifolia*) exhibit high locality and stable genetic characteristics, ensuring population continuation and adaptation to the harsh environment (Knief et al., 2010). The colonization of phyllosphere microorganism communities is significantly influenced by plant genotypes (Larran et al., 2002; Upper et al., 2003), demonstrating the intricate association between phyllosphere bacteria and fungi influenced by factors such as scale, background dependence, and environmental conditions.

Moreover, it revealed a notable decrease in nodes, edges, and average degree of phyllosphere bacteria during spring, while those of fungi were at their highest. This strengthens the evidence supporting niche complementary effects (Fuhrman, 2009; Chen et al., 2022). During the germination and growth of desert deep-rooted plants in spring, phyllosphere microorganisms exhibited a strong niche complementary effect, particularly due to the presence of saprophytic fungi that supply ample nutrients (Fierer and Jackson, 2006; Bergelson et al., 2019). This symbiosis enhances the adaptability of desert plants to challenging environmental conditions during the summer (Hernandez et al., 2021; Chen et al., 2022).

The population size and diversity of phyllosphere bacteria and fungi exhibit associations with leaf position, plant structure, canopy height, plant genotypes, leaf morphological characteristics, physicochemical properties, climatic factors, and geographical differences (Oliveira et al., 1991; Jacques et al., 1995; Larran et al., 2002; Lindow and Brandl, 2003; Upper et al., 2003; Schreiber et al., 2005). Microbial abundance positively correlates with multi-functionality, influencing diverse ecosystem functions (Chen et al., 2022). The investigation demonstrated that the diversity of phyllosphere bacteria is influenced by specific leaf area and temperature, while the diversity of phyllosphere fungi is impacted by leaf electrical conductivity, organic carbon, and horizontal radiation.

However, it found that the significant direct impact of leaf morphological characteristics on the multi-functionality of phyllosphere bacteria, surpassed the effects of climate and leaf physicochemical properties (Lindow and Brandl, 2003; Schreiber et al., 2005). Similarly, leaf morphological characteristics and physicochemical properties have a greater direct effect on the multi-functionality of phyllosphere fungi compared to climate (Kembel et al., 2014; Zhang et al., 2022). Nonetheless, the indirect and total effects of climate on multi-functionality surpass those of leaf characteristics and physicochemical properties, emphasizing the substantial influence of environmental factors (Beattie, 2011; Qian et al., 2020).

The study highlights the importance of the physical and chemical properties of leaves in determining the stability of multi-functionality in phyllosphere microorganisms’ ecosystems (Yadav et al., 2005; Beattie, 2011; Liu et al., 2023; Luo et al., 2023). Notably, the multi-functionality of phyllosphere microorganisms, including bacteria and fungi, showed no notable correlation with climatic conditions (Wei et al., 2022). The findings underscore the complex interplay of environmental factors, plant morphology, and microbial diversity, emphasizing their profound implications for the stability and functionality of phyllosphere microorganism communities (Stapleton and Simmons, 2006; Postiglione et al., 2022).

## 5 Conclusion

During spring, phyllosphere bacteria exhibited their lowest diversity index, multi-functionality, nodes, edges, average degree, and positive/negative correlations between edges, while fungi displayed peak values. Despite a pronounced niche complementary effect between bacteria and fungi, phyllosphere bacteria demonstrated a more intricate network structure in various seasons or geographical locations. Notably, the multi-functionality of phyllosphere microorganisms correlated significantly with overall potassium content, and the multi-functionality of phyllosphere fungi was linked to leaf electrical conductivity. Across three regions, phyllosphere bacteria showed relative aggregation across seasons, while fungi exhibited relative scattering. Leaf morphological characteristics and climatic factors had a greater impact on phyllosphere fungi abundance than on bacteria. The indirect and total effects of climate on multi-functionality surpassed leaf physicochemical properties and morphological characteristics. Specifically, the direct effect of leaf morphological characteristics on phyllosphere bacteria’s multi-functionality exceeded that of climate. Physicochemical properties and leaf morphology directly influenced phyllosphere fungi multi-functionality. The observed niche complementary effect in phyllosphere microorganisms (bacteria and fungi) is crucial for desert ecosystem stability and functionality. Understanding these community structure differences is scientifically significant for the protection and restoration of desert ecosystems.

## Data availability statement

The original contributions presented in the study are included in the article/Supplementary material, further inquiries can be directed to the corresponding author.

## Author contributions

YZ: Data curation, Software, Writing – original draft. YD: Data curation, Writing – original draft. ZZ: Conceptualization, Supervision, Writing – review & editing. WI: Conceptualization, Supervision, Writing – review & editing. FZ: Conceptualization, Funding acquisition, Project administration, Supervision, Writing – review & editing.

## Footnotes

^1^https://gephi.org/ (accessed on: 15 November 2023).
