# Supplementary material for: Unveiling the diversity, composition, and dynamics of phyllosphere microbial communities in Alhagi sparsifolia across desert basins and seasons in Xinjiang, China
Source: Front Microbiol. 2024 Mar 25;15:1361756. doi: 10.3389/fmicb.2024.1361756 (PMC10999668; doi:10.3389/fmicb.2024.1361756)
Supplement: Supplementary file 1 [file Table_1.DOCX]

**Table S1** Effects of seasons on physicochemical properties and morphological characteristics of leaves

| **Species** | **Site** | **Season** | **pH** | **LOC (g/kg)** | **TN (g/kg)** | **TP (g/kg)** | **TK (g/kg)** |
| --- | --- | --- | --- | --- | --- | --- | --- |
| *A. sparsifolia* | CL | **SP** | 6.46±0.03c | 434.31±3.23a | 28.04±0.33a | 1.11±0.01a | 15.74±0.24a |
|  |  | **SU** | 6.88±0.04a | 433.70±2.27a | 20.14±0.11b | 0.74±0.01c | 14.48±0.18b |
|  |  | **AU** | 6.74±0.04b | 409.63±3.45b | 20.66±0.10b | 0.79±0.01b | 15.17±0.09a |
|  | MSW | **SP** | 6.61±0.05b | 443.23±4.45a | 33.40±0.73a | 1.62±0.02a | 12.76±0.39a |
|  |  | **SU** | 6.47±0.03b | 425.30±3.69b | 23.43±0.12b | 0.93±0.01b | 5.57±0.08b |
|  |  | **AU** | 6.87±0.05a | 432.51±3.95ab | 19.54±0.15c | 0.77±0.02c | 12.74±0.06a |
|  | TLF | **SP** | 6.44±0.08b | 467.02±6.13a | 31.26±0.89a | 1.61±0.02a | 8.65±0.10b |
|  |  | **SU** | 6.77±0.05a | 441.77±1.73b | 20.96±0.17c | 0.90±0.01c | 9.48±0.06a |
|  |  | **AU** | 6.70±0.08a | 435.70±3.16b | 25.22±0.29b | 1.19±0.01b | 8.84±0.02b |

| **Species** | **Site** | **Season** | **EC (μs/cm)** | **LAR (cm^2^)** | **LDW (g)** | **SLA (cm^2^/g)** | **SLW (g/cm^2^)** |
| --- | --- | --- | --- | --- | --- | --- | --- |
| *A. sparsifolia* | CL | **SP** | 1762.25±130.62b | 1.76±0.06a | 0.02±0.0003c | 98.40±2.89a | 0.01±0.0002c |
|  |  | **SU** | 3235.00±145.75a | 2.08±0.05a | 0.03±0.0008b | 75.93±3.24b | 0.01±0.0005b |
|  |  | **AU** | 1695.00±59.23b | 2.11±0.25a | 0.03±0.002a | 64.75±3.27c | 0.02±0.0008a |
|  | MSW | **SP** | 75.5±5.54b | 1.15±0.10b | 0.01±0.0009b | 98.14±8.24a | 0.01±0.0008b |
|  |  | **SU** | 2575.00±117.65a | 2.15±0.10a | 0.03±0.0006a | 74.51±4.51b | 0.01±0.0008a |
|  |  | **AU** | 72.48±5.32b | 2.21±0.20a | 0.03±0.0008a | 71.82±5.06b | 0.01±0.0009a |
|  | TLF | **SP** | 149.65±10.88b | 0.49±0.05b | 0.01±0.0005c | 94.46±3.17a | 0.01±0.0003c |
|  |  | **SU** | 3125.00±153.70a | 0.82±0.07a | 0.01±0.0002b | 76.11±6.36b | 0.01±0.001b |
|  |  | **AU** | 143.66±10.45b | 0.86±0.02a | 0.02±0.0003a | 50.08±0.96c | 0.02±0.0004a |

**Note:** **CL**, Cele; **MSW**, Mosuowan; **TLF**, Turpan; **SP**, spring; **SU**, summer; **AU**, autumn; **EC**, electrical conductivity; **LOC**, leaf organic carbon; **TN**, total nitrogen; **TP**, total phosphorus; **TK**, total potassium; **LAR**, leaf area; **LDW**, leaf dry weight; **SLA**, specific leaf area; **SLW**, specific leaf weight. Different lowercase letters (a, b, and c) indicate that the same site and plants and different seasons have significant differences (LSD test, *P* < 0.05).

**Table S2** Three typical regional spring, summer and autumn climate factors in 2022 year (mean)

| **Site** | **Season** | **Atm (hPa)** | **WS (m/s)** | **WD (°)** | **DR (w/m^2^)** | **SR (w/m^2^)** | **HR (w/m^2^)** | **Hum (%)** |
| --- | --- | --- | --- | --- | --- | --- | --- | --- |
| CL | **SP** | 854.99 | 4.13 | 213.63 | 288.37 | 94.73 | 306.46 | 26.20 |
|  | **SU** | 851.74 | 3.46 | 195.02 | 293.75 | 92.55 | 307.92 | 23.66 |
|  | **AU** | 857.97 | 3.06 | 208.06 | 308.12 | 63.34 | 253.50 | 24.58 |
| MSW | **SP** | 969.79 | 4.21 | 186.71 | 261.38 | 99.84 | 281.16 | 27.64 |
|  | **SU** | 964.89 | 4.04 | 199.54 | 249.55 | 107.84 | 284.32 | 27.36 |
|  | **AU** | 973.43 | 3.38 | 169.83 | 239.25 | 64.30 | 197.62 | 31.99 |
| TLF | **SP** | 1011.62 | 2.37 | 196.76 | 252.62 | 103.63 | 281.20 | 20.76 |
|  | **SU** | 1005.56 | 2.49 | 203.89 | 238.97 | 110.22 | 281.27 | 23.27 |
|  | **AU** | 1014.75 | 1.68 | 197.45 | 295.64 | 63.48 | 228.22 | 21.45 |

**Note:** **CL**, Cele; **MSW**, Mosuowan; **TLF**, Turpan; **SP**, spring; **SU**, summer; **AU**, autumn; **Atm,** atmospheric pressure; **WS,** wind speed; **WD,** wind direction; **DR,** direct radiation; **SR,** scattered radiation; **HR,** horizontal radiation; **Hum,** humidity.

**Table S3** Network co-occurrence properties of phyllospheric microganism (bacteria and fungi) in different seasons and geographical locations.

|  | **Season/Site** | **Positive edge** | **Negative edge** | **Clustering coefficient** | **Average path length** | **Centralization closeness** | **Centralization betweenness** | **Edge connectivity** | **Average neighborhood** | **Connectance** | **Nestedness** |
| --- | --- | --- | --- | --- | --- | --- | --- | --- | --- | --- | --- |
| Bacteria | **SP** | 285 | 566 | 0.99 | 1.01 | 0.02 | 4.09E-05 | 36 | 41.57 | 0.99 | 0.26 |
|  | **SU** | 1070 | 1577 | 0.98 | 1.02 | 0.04 | 6.31E-05 | 58 | 72.57 | 0.98 | 0.42 |
|  | **AU** | 672 | 1169 | 0.98 | 1.03 | 0.05 | 0.000104677 | 49 | 60.42 | 0.97 | 0.37 |
|  | **CL** | 305 | 608 | 0.97 | 1.03 | 0.06 | 0.000234989 | 35 | 42.50 | 0.97 | 0.40 |
|  | **MSW** | 1077 | 1577 | 0.98 | 1.02 | 0.03 | 4.63E-05 | 59 | 72.76 | 0.98 | 0.42 |
|  | **TLF** | 497 | 1012 | 0.98 | 1.02 | 0.04 | 8.00E-05 | 45 | 54.89 | 0.98 | 0.35 |
|  |  |  |  |  |  |  |  |  |  |  |  |
| Fungi | **SP** | 114 | 71 | 0.66 | 1.47 | 0.65 | 0.09 | 6 | 14.70 | 0.53 | 0.66 |
|  | **SU** | 46 | 46 | 0.85 | 1.23 | 0.38 | 0.04 | 6 | 12.50 | 0.77 | 0.80 |
|  | **AU** | 24 | 19 | 0.74 | 1.35 | 0.54 | 0.11 | 3 | 8.17 | 0.65 | 0.81 |
|  | **CL** | 76 | 67 | 0.87 | 1.16 | 0.28 | 0.01 | 10 | 16.05 | 0.84 | 0.78 |
|  | **MSW** | 46 | 46 | 0.85 | 1.23 | 0.38 | 0.04 | 6 | 12.50 | 0.77 | 0.80 |
|  | **TLF** | 40 | 57 | 0.80 | 1.29 | 0.45 | 0.05 | 4 | 12.41 | 0.71 | 0.81 |

**Note:** **CL**, Cele; **MSW**, Mosuowan; **TLF**, Turpan; **SP**, spring; **SU**, summer; **AU**, autumn.
